# Intranuclear birefringent inclusions in paraffin sections by polychromatic polarization microscopy

**DOI:** 10.1038/s41598-021-85667-8

**Published:** 2021-03-18

**Authors:** Aiste Vitkunaite, Aida Laurinaviciene, Benoit Plancoulaine, Allan Rasmusson, Richard Levenson, Michael Shribak, Arvydas Laurinavicius

**Affiliations:** 1 National Center of Pathology, Affiliate of Vilnius University Hospital Santaros Clinics, Vilnius, Lithuania; 2 Institute of Biomedical Sciences, Faculty of Medicine, Vilnius University, Vilnius, Lithuania; 3 ANTICIPE, Inserm (UMR 1086), Cancer Center F. Baclesse, Normandy University, Caen, France; 4 Department of Pathology and Laboratory Medicine, UC Davis Health, Sacramento, CA USA; 5 Marine Biological Laboratory of University of Chicago, Woods Hole, MA USA

**Keywords:** Cancer imaging, Nuclear organization

## Abstract

Intranuclear birefringent inclusions (IBI) found in various cell types in paraffin-embedded tissue sections have long been considered to be a tissue processing artifact, although an association with biological processes has been suggested. We applied polychromatic polarization microscopy to image their spatial organization. Our study provides evidence that IBI are caused by liquid paraffin-macromolecular crystals formed during paraffin-embedding procedures within cells and potentially reflect an active transcriptional status.

## Introduction

The phenomenon of intranuclear birefringent inclusions (IBI) under polarized light in paraffin sections was reported in 1951 by Nedzel^1^, who described it as an artifact to be avoided in studies based on polarized light. The IBI were thought to have originated from residual paraffin due to insufficient deparaffinization and clearing during the tissue processing; heating the section under polarized light revealed loss of the signal at the paraffin melting temperature. In 1968, Vlachos suggested that the chemical nature and physical state of nuclear macromolecules, especially DNA, could act as a substrate to selectively bind paraffin molecules^2^. They demonstrated that tissue preparation steps such as deparaffinization, hydration and dehydration were important for nuclear birefringence. IBI has also been noted in lemon fruit explant cells cultured in vitro and imaged using polarized light and phase-contrast, but the signal was thought to depend on a filamentous nucleolar component^3^. Other studies have linked nuclear birefringence with lymphocyte activation in rat spleen cell suspensions stained with neutral red^4^ and with liquid crystalline chromosome formations noted in dinoflagellate cultures^5^.

Nuclear morphology can reflect states of chromatin dynamics and cell signal transduction^6^, and it is reasonable to find that new imaging modalities can provide novel digital signatures of nuclear properties. DNA condensation and epigenetic regulation of chromatin structures, via mechanisms linked to macromolecular crowding in the nuclei and more complex packing, can be related to transcriptional activity^7,8^. Additional non-physiological conformational changes in the nuclear components may also be introduced during tissue fixation and paraffin embedding procedures^9,10^. Due to these changes in conformational state of nonhistone proteins, Freivalds associated cell nuclear optical anisotropy to differences in the chromatin stereo-arrangement and suggested that IBI could be a useful method for investigating cell nucleus functional states in relation to the prognosis of patients with cancer^11^. Recently, chromatin compartmentalization has been explained by liquid–liquid phase separation induced by a variety of genetic processes^12^. These studies suggest that IBI may be related to chromatin conformational changes and regulatory mechanisms in the nucleus. We therefore explored this phenomenon using the technique of polychromatic polarization microscopy (PPM), which we found to provide strong and distinct signals for IBI.

## Materials and methods

### Microscope set up

Polychromatic polarization micrographs of IBI were acquired using an *Olympus BX63* microscope equipped with a *DP80* camera and a custom spectral polarization state generator and analyzer (Supplementary Fig. 1). This enabled observation of birefringent structures with the benefit that the observed colors directly report the orientation of the birefringence axis of the structures inside the specimen^13^. For the temperature-sensitivity experiments, a heating plate connected to a *Haltronic HPS305D* switching power supply was used. The entire microscope set up was managed using the *CellSens* software which was also used to acquire and save the resulting images. The settings used for particular image acquisitions are provided in the relevant figure legends.

### Observation of IBI in FFPE and frozen tissue sections

We studied 59 4-µm thick formalin-fixed, paraffin-embedded (FFPE) deparaffinized unstained tissue sections coverslipped with aqueous mounting media (Kaiser’s glycerol gelatin, *Merck*) from surgical excision samples (neoplastic and non-neoplastic kidney, liver, stomach, colon, thyroid, lung, endometrium tissues) from 20 patients. 4-µm-thick FFPE tissue sections were cut on a microtome, put on *TOMO* adhesion microscopy slides (*Matsunami Glass USA Inc.*) and dried at room temperature. Deparaffinization used routine protocols with solutions of xylene, isopropyl and ethyl alcohols. After deparaffinization, sections were washed in distilled water and coverslipped with aqueous mounting media (Kaiser’s glycerol gelatin, *Merck*). The number of IBI signals was assessed semi-quantitatively by PPM: “+” indicated just a few IBI in the whole tissue section and “+++” an abundant amount of IBI.

An additional 89 frozen tissue sections from 37 cases were also investigated. 5-µm-thick frozen tissue sections were cut and put on *TOMO* adhesion microscope slides (*Matsunami Glass USA Inc.*) and stained using a routine hematoxylin–eosin protocol for frozen sections. The sections were coverslipped with aqueous mounting media (Kaiser’s glycerol gelatin, *Merck*) and immediately investigated for IBI signals.

All tissue samples originated from the Lithuanian National Center of Pathology and the study was performed under permission of Vilnius Regional Biomedical Research Ethics Committee No. 2019/6-1148-637.

### pH dependence

Using an *inoLab WTW* pH measuring device, we prepared 9 solutions (pH 2, 3, 4, 5, 6, 7, 8, 9 and 10) from distilled water by adding NaOH or HCl solutions. 4-µm-thick deparaffinized FFPE tissue sections were immersed for 1 min in aqueous solutions for each pH value. The same regions of interest were observed before and after the treatment with different pH solutions using PPM. From the captured images it was possible to objectively evaluate the intensity of IBI signals, compare IBI color composition and count the number of IBI. Each part of the experiment was imaged with *Olympus BX63* microscope equipped with the PPM device, DP80 camera and *LUCPlanFLN 40* × objective.

### Enzymatic digestion

RNase and DNase enzymes were used from *Invitrogen by Thermo Fisher Scientific RecoverAll Kit* which is used for RNA purification. Proteinase was used from *Cobas DNA Sample Preparation Kit*, typically used for DNA extraction from FFPE tissue sections. Four serial sections were cut, and deparaffinized. Three of the sections were subsequently treated with 30 µl of each enzyme solutions, coverslipped with mounting glass and sealed with gum glue to ensure that the mixture did not evaporate. Each enzyme solution mix was prepared according to the manuals from the kits: proteinase solution mix—180 µl tissue lysis buffer, 70 µl proteinase K, incubation at 56 °C temperature for 1 h; DNase solution mix—4 µl DNase, 6 µl 10xbuffer, 50 µl water, incubation for 30 min. at room temperature; RNase solution mix—10 µl RNase, 50 µl water, incubation for 30 min. at room temperature. After incubation the gum glue was removed and sections were washed with distilled water, stained with Hoechst solution (500 µg/ml) for fluorescent nuclei visualization and coverslipped with aqueous mounting media (Kaiser’s glycerol gelatin, *Merck*). The same region from all four serial sections was imaged by PPM microscopy to compare the three enzymatically treated sections with the fourth treated with deparaffinization only.

### Melting and recrystallization experiment

For this experiment we used 4-µm-thick hepatocellular carcinoma tissue sections deparaffinized and coverslipped with aqueous mounting media (Kaiser’s glycerol gelatin, *Merck*). The prepared samples were placed in the heating plate and imaged before heating. During the experiment the temperature was registered manually using an infrared thermometer *UNI-T UT300A* at fixed time intervals and the first and last appearance of the IBI were recorded. The power supply for the heating plate was switched off when all of the IBI had disappeared. The IBI recrystallized during the cooling process at room temperature, and the time was noted when the first and last IBI recrystallized. The same region was imaged after the recrystallization to reveal any changes in the structural and signal properties of IBI. The experiment was repeated for ten cycles with a 5-min gap between each cycle to allow the specimen to cool down to 30 °C.

To compare the images taken after each heating cycle and the structure of IBI, we calculated the area and perimeter of IBI in the 14 selected nuclei in all acquired images. The area and perimeter were evaluated based on number of segmented pixels, the shape factor was calculated from the area and perimeter ratio based on the formula $\frac{4\pi a}{{p}^{2}}$, where a—area; p—perimeter. Images were analysed with *ImageJ*, and the extracted information exported to *MS Excel* for further calculations and visualization.

### Immunohistochemistry studies

Immunohistochemistry (IHC) testing was performed to characterize cell types and their biological properties in combination with the PPM imaging data to explore potential associations of the IBI. Identification of specific cell types and biological activity markers was performed based on IBI overlap with the IHC markers in the same cells using PPM and bright field microscopy. Routine IHC staining protocols were optimized to obtain both IHC and IBI signals from the sections. The optimization included drying of tissue sections after placement on the adhesion slide, antigen retrieval methods, changes in section thickness, concentrations of antibodies, type of coverslipping media. The strength of association between certain IHC marker-positive nuclei and IBI signals was assessed by observing double-positive nuclei showing both specific marker expression and IBI.

The exhaustive list of antibodies, dilutions and detection systems used was: anti-CD3 (*A0452, rabbit polyclonal antibody, 1:150, Dako*), anti-CD20 (*M0755, mouse monoclonal antibody, clone L26, 1:500, Dako*), anti-CD57 (*M7271, mouse monoclonal antibody, clone TB01, 1:50, Dako*), anti-CD68 (*M0876, mouse monoclonal antibody, clone PG-M1, 1:100, Dako*), anti-HEPA (*M7158, mouse monoclonal antibody, clone OCH1E5, 1:500, Dako*), anti-P53 (*M7001, mouse monoclonal antibody, clone DO-7, 1:200, Dako*), anti-PAX2 (*ab79389, rabbit monoclonal antibody, clone EP3251, 1:900, Abcam*), anti-PAX5 (*PAX5-L-CE, mouse monoclonal antibody, clone 1EW, 1:100, Leica Biosystems*), anti-PAX8 (*363M-16, mouse monoclonal antibody, clone MRQ-50, 1:150, CellMarque*) used with *Dako EnVision FLEX* detection system; anti-Ki67 (*M7240, mouse monoclonal antibody, clone MIB-1, 1:200, Dako*), anti-HIF1α (*AC-0108, rabbit monoclonal antibody, clone EP118, 1:200, Epitomics*), anti-ATRX (*ab188027, mouse monoclonal antibody, clone CL0537, 1:300, Abcam*) used with *Ventana BenchMark UltraView DAB* detection system; anti-TTF1 (*rabbit monoclonal antibody SP141, ready to use, Ventana*) used with *Ventana OptiView DAB* detection system.

### IBI association with transcription factor TFIIE activity

Recombinant anti-TFIIE alpha/GTF2E1 antibody (*ab140634, rabbit monoclonal antibody, 1:100, Abcam*) was used for the IHC stain. The best results for this marker were achieved using the *Ventana BenchMark UltraView Universal Alkaline Phosphatase Red* detection kit. To investigate the association between the transcription activity factor TFIIE marker and the IBI signals we used 4 renal cell carcinoma and 4 hepatocellular carcinoma cases previously identified to be rich in IBI signals. From each case we prepared 4-µm-thick tissue sections, deparaffinized them before staining with Hoechst solution (1 µg/ml) for 1 min and then washed with distilled water. From each case 20 representative fields of view were imaged using an *Olympus LUCPlanFLN 20* × objective. Overall, three sets of images were acquired: (1) using PPM; (2) fluorescent imaging with *Olympus U_FUNA* filter cube for Hoechst-stained nuclei visualization superimposed with IBI signals. The third (3) set of images was acquired from the same 20 fields from slides re-stained with THIIE IHC bright field microscopy. Nuclei stained with red Fast Red/Naphthol chromogen from the *Ventana* detection kit were identified as positive for TFIIE marker expression. Image data of the red channel were extracted from the bright field images of the TFIIE-stained slides to generate a mask of TFIIE-positive nuclei. Finally, the corresponding fields of view from all three image sets were registered to visualize and enumerate the nuclei stained with Hoechst in blue, IBI signals from PPM in pink, and TFIIE nuclei in green masks.

## Results

### Localization, structure and distribution of IBI

**Figure 1:**
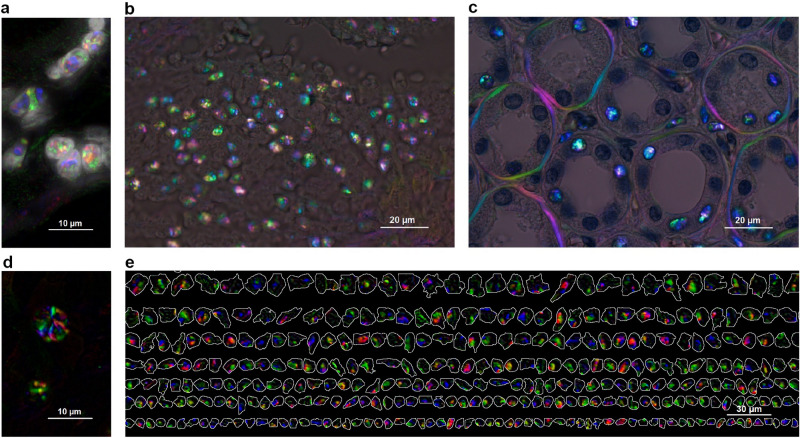
Spatial patterns of IBI captured by PPM. (**a**) Extracted PPM signals of intranuclear inclusions overlaid with Hoechst-stained fluorescence image for nuclei visualization; (**b**) endometrioid carcinoma tissue section by PPM after deparaffinization showing a cluster of IBI; (**c**) IBI and basement membrane collagen PPM signals in kidney tissue section stained with hematoxylin for nuclei visualization and imaged using PPM; (**d**) Crystalloid structure of the IBI—nuclear inclusions captured in FFPE unstained tissue sections imaged by PPM with digital background correction; (**e**) gallery of nuclei segmented from Hoechst-stained renal cell carcinoma tissue imaged by fluorescence and overlaid with IBI acquired by PPM. All images acquired with *Olympus BX63* microscope equipped with an *Olympus DP80* camera using the *CellSens* acquisition software. Objectives: (**b**,**c**) *Olympus UPlanApo 100x*, (**a**,**d**) additional ×2 magnification lens used, (**e**) *Olympus LUCPlanFLN 20x*, with *Olympus U_FUNA* DAPI fluorescence filter cube. Original images acquired at a resolution of 1360 × 1024 px, resized and put into multi-panel figure using the *GIMP* image editor.

Compared to conventional polarization microscopy we found that PPM generates strong and distinct imaging signals that highlight the spatial organization of the IBI: the PPM imaging pattern discloses randomly organized compartments inside the crystalloid structures it detects (Fig. 1). Distinct colors of the nuclei compartments represent different refractive angles of IBI structural components that reflect relevant stereo-arrangements. Figure 1c furthermore illustrates that the IBI signals in the nuclei do not overpower more traditional polarization information, for example, from collagen. In all tissues examined, IBI were detected exclusively within nuclei while their size corresponded to the size of the nuclei (Fig. 1a,c,e). The imaging properties of the IBI were also explored using quantitative orientation-independent differential interference contrast (OIDIC), phase contrast and fluorescence microscopy (Supplementary Figs. 2, 3). Application of different tissue imaging techniques enabled comparison of the properties of the IBI structure and visualization of observed signals using different microscopy methods which confirms that IBI signals are not only visible under polarized light.

We studied FFPE deparaffinized tissue sections from different human organs samples using PPM (Supplementary Fig. 4). IBI were detectable in 48 patient cases out of 59 of samples in variable frequencies, exclusively in the nuclei of the epithelial, interstitial or endothelial cells, further confirmed by histology and relevant IHC markers. Interestingly, clusters of IBI-containing inflammatory infiltrates were frequently observed in the peritumoral tissue near tumor margins (Fig. 1b). This indicated that IBI signals can be found in the different tissue types examined and are not distributed accidentally across the tissue slide. In contrast, IBI were not found after exhaustive search in 89 frozen tissue sections.

### Physical and chemical properties of IBI

To further explore the properties of IBI a series of experiments was performed. IBI PPM signals were detectable within a range of 2–10-µm tissue section thickness slides and their frequency directly correlated to section thickness (Supplementary Fig. 5). We did not find any changes in the IBI while varying the pH value from 2 to 10 of the aqueous solutions applied to the sections. Additionally, enzymatic treatment with RNase, DNase or proteinase was not found to influence the IBI signals. The resistance of the IBI signal to the enzyme digestion and pH modulation suggests that the birefringent substance is not of biomolecular origin but is likely to be represented by less sensitive substances such as hydrocarbons (paraffin). In contrast, the signals did deteriorate and were finally lost after thermal antigen retrieval before IHC procedures, intense dehydration steps before coverslipping and/or after extended immersion in xylene solution, including coverslipping with xylene-based mounting media.

Varying heating conditions revealed that IBI in coverslipped tissue sections mounted with aqueous glycerin media disappeared at temperatures higher than 50–53 °C, which corresponds to the paraffin melting point. However, they would reappear in the same nuclei after cooling the slide down to temperatures in the range of 37–47 °C suggesting that the main ingredient of IBI signal is polarization of the paraffin crystal structure which is melted when signal disappears and recrystallizes again causing the effect of IBI. (Supplementary Video 1, Supplementary Table 1). We repeated 10 heating/cooling cycles in the same tissue area using a heated microscope stage which revealed a striking persistence of the IBI reassembling in the same nuclei without notable loss of PPM signal intensity or IBI size; even so, the shape and distribution of the IBI color compartments reappeared in a seemingly random fashion (Supplementary Fig. 6). This suggests a strong molecular bond between melted liquid paraffin and the nuclear components whereas the paraffin crystal reappearance results in random IBI shapes. Importantly, the IBI reappearance was immediately disrupted if the heated tissue sections were allowed to dry out or were dehydrated at the temperatures above the paraffin melting point, indicating the importance of water in maintaining the molecular bonds.

### Association between biological markers and IBI

**Figure 2:**
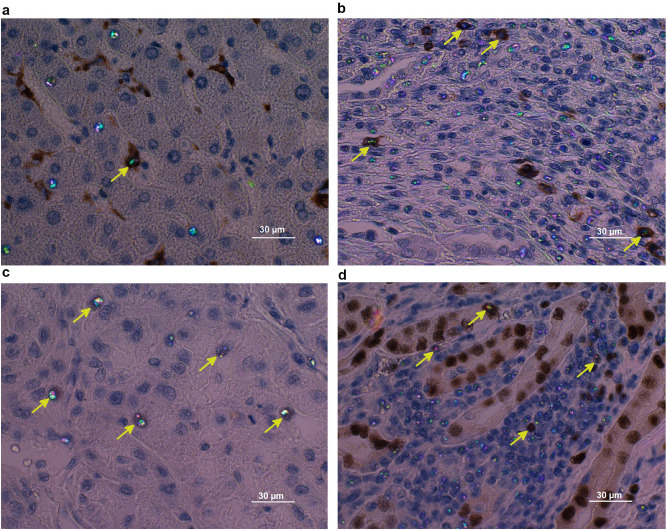
IHC studies to identify IBI-positive cell types and biological processes: (**a**) IBI present in CD68-positive macrophages (arrow) but also in CD68-negative cells (hepatocellular carcinoma); (**b**) IBI present in CD20-positive B cells (arrow) but also in CD20-negative cells (renal cell carcinoma); (**c**) PAX2 marker-specific B cells transcription factor—on renal cell carcinoma tissue section; (**d**) PAX8 marker-specific renal cancers transcription factor—on renal cell carcinoma tissue section. All images acquired with *Olympus BX63* microscope, *DP80* camera, objective *Olympus Ach 60* × and PPM modality. Original images acquired at a resolution of 1360 × 1024 px, resized and put into multi-panel figure using the *GIMP* image editor.

The IBI could be detected in different cell types positive for CD3, CD20, CD57, CD68, HEPA IHC markers (Fig. 2a,b). IBI were not associated with proliferation (Ki67), angiogenesis (hypoxia-inducible factor 1α), chromatin remodeling factor (ATRX) or cellular tumor antigen P53. Associations of different degrees were found with tissue-specific PAX2, PAX5, PAX8, and TTF1 transcription factors (Fig. 2c,d). The most prominent overlap (ranging from 5 to 90% of IBI were detected within general transcription-factor-TFIIE-positive cells) as found in liver and kidney tissues (Supplementary Fig. 7, Supplementary Table 2). Although we were not able to obtain strong evidence for consistent association between the transcription IHC markers and IBI, this partial correlation may be explained by cell/tissue specificity of the IHC markers as well as possible differences in the dynamics of the specific transcriptional protein expression and the macromolecular nucleoprotein conformations revealed by the IBI phenomenon.

## Discussion

Our study demonstrates the utility of PPM imaging for generating strong and distinct signals for detection of IBI in FFPE tissue sections. This technique allowed us to explore both physical and chemical properties of the IBI as well as their potential biological associations. Our results are in line with the previous findings^1^ that the IBI phenomenon is produced by birefringence of paraffin remaining in the nuclei of some cells after routine tissue processing by paraffin technique. Remarkably, the IBI were observed exclusively in nuclei of FFPE-processed tissues and were never found in frozen tissue sections. Repeated melting-recrystallization experiments revealed that the IBI signal reappeared consistently in the same nucleus after each cycle, while the formation of IBI signals was disrupted entirely if the sections were allowed to dry out above the paraffin melting point. This is indicative of strong water-dependent bonds between melted paraffin and the nuclear components in some cells. We further explored the IBI associations with IHC biomarkers in surgical excision samples and obtained evidence for potential association of the IBI phenomenon with high transcriptional activity in the nuclei, in agreement with the previous studies that it could be useful method for the cell nucleus functional state investigation^11^.

The IBI structural pattern, visualized by PPM as birefringence, is caused by long straight-chain paraffin molecules that become tangled and form crystals below the melting point. The paraffin molecules form a lattice of strongly birefringent crystals which usually appear as needles, plates, and malcrystalline forms^14^. An aggregate of paraffin crystals in a nucleus exhibits the IBI pattern depicted in Fig. 1. The IBI signal did not notably deteriorate after multiple melting cycles; however, it was entirely lost if the water-dependent bond of melted paraffin and nuclear components was disrupted by drying of the section above the paraffin melting point. This is suggestive of a peculiar hydrophilic macromolecular state of the individual nuclei, resistant to routine dehydration procedures in tissue processing. This further raises a question of potential functional implications of the nuclear properties.

We investigated the potential biological significance of the IBI in surgical excision samples of neoplastic and non-neoplastic tissues. The IBI was found with variable frequency in different tissues and in various cell types—neoplastic and non-neoplastic epithelium and interstitial cells as well as peritumoral lymphocytic infiltrates. Our IHC studies did not reveal notable associations of IBI to any cell type or status (proliferation, apoptosis) except transcription activation factor TFIIE, a biomarker of general (cell-type independent) transcriptional activity^15^. This is in line with previous observation by Surjan that the birefringence of nuclear neutral red stain was increased during lymphocyte activation due to gene activation and change of cytochemical properties^4^. Conformational properties of euchromatin and/or other macromolecular transformations related to active gene transcription processes could be responsible for formation of strong hydrophilic bonds with liquid paraffin molecules during the tissue processing. Of note, models of regulatory subnuclear chromatin compartmentalization by liquid–liquid phase separation and protein-chromatin bonds could provide further insights into the phenomenon of IBI^12^.Reference (14-18) is not cited in text. Please either cite it or delete this reference from the reference list.

In conclusion, we propose PPM as an imaging technique that generates strong signals of IBI and highlights their spatial organization. Our study supports the evidence that IBI represent a tissue processing consequence of paraffin-embedding techniques and are possibly related to transcriptional activity of individual cells possessing nuclear properties responsible for strong molecular bonds with liquid paraffin wax.

## Supplementary information

Supplementary information.

Supplementary video 1.
