# Supplementary material for: Intranuclear birefringent inclusions in paraffin sections by polychromatic polarization microscopy
Source: Sci Rep. 2021 Mar 18;11:6275. doi: 10.1038/s41598-021-85667-8 (PMC7973427; doi:10.1038/s41598-021-85667-8)
Supplement: Supplementary file 1 — Supplementary information. [file 41598_2021_85667_MOESM1_ESM.pdf]

INTRANUCLEAR BIREFRINGENT INCLUSIONS IN PARAFFIN SECTIONS BY POLYCHROMATIC POLARIZATION MICROSCOPY

Aiste Vitkunaite[1], Aida Laurinaviciene[1,2], Benoit Plancoulaine[2,3], Allan Rasmusson[1,2], Richard Levenson[2,4], Michael Shribak[2,5], Arvydas Laurinavicius[1,2*]

[1]National Center of Pathology, Affiliate of Vilnius University Hospital Santaros Clinics, Vilnius, Lithuania

[2] Institute of Biomedical Sciences, Faculty of Medicine, Vilnius University, Vilnius, Lithuania

[3]ANTICIPE, Inserm (UMR 1086), Cancer Center F. Baclesse, Normandy University, Caen, France

[4]Dept. of Pathology and Laboratory Medicine, UC Davis Health, Sacramento, CA, USA

[5]Marine Biological Laboratory of University of Chicago, Woods Hole, MA, USA

Contact information: arvydas.laurinavicius@vpc.lt

Second contact information: mshribak@mbl.edu

Supplementary Figure 1.

An optical scheme of the polychromatic polarization microscope is shown in [1]. PPM is available from Marine Biological Laboratory (Woods Hole, USA) for dissemination to other users and can be manufactured per request (https://www.mbl.edu/bell/current-faculty/shribak-lab/).

A conventional polarized light microscope uses scalar white beam interference, which displays objects in Newton's interference colors when one of the interfering beams is retarded relative to the other by the optical path difference (retardance) from 400nm to 2000nm. However, most biological structures have retardance value less than 100nm. Therefore, biological birefringent structures under a standard polarized light microscope appear gray, with contrast disappearing at certain orientations. The PPM exploits a vector interference of two white light beams. Unlike the standard microscope, the full spectrum interference colors in PPM appear even in specimens with low retardance levels of only a few nanometers, which was not possible before. As the beam passes through a transparent biological specimen, its birefringent structures modify the beam's polarization in such way that the non-birefringent specimen parts appear gray and the birefringent structures appear colored. The hue of the birefringent structure indicates its slow axis orientation, and the brightness of the structure is proportional to its retardance.

The PPM is based on a standard polarization microscope with white light illumination, which is equipped with a special polychromatic polarization state generator and achromatic circular analyzer. The polarization state generator produces polarized light with the polarization ellipse orientation determined by the wavelength, which we call the spectral polarization fan. An example of the fan with the right polarization ellipses for visible spectrum from 440nm to 660nm by polychromatic polarization state generator is shown in part (a). The major axis of red polarization ellipse (λ=660nm) is oriented along the initial axis (ψ=0°). Then the major axes of orange polarization ellipse (λ=609nm), yellow polarization ellipse (λ=566nm), green polarization ellipse (λ=528nm), cyan polarization ellipse (λ=495nm) and blue polarization ellipse (λ=466nm) are oriented at 30°, 60°, 90°, 120° and 150° to the initial axis, correspondently. All polarization ellipses have the same ellipticity angle ε~40° [2].

If the specimen under investigation isn't birefringent then the beam passes through it without alteration of the polarization. The left circular analyzer evenly transmits all wavelengths, and the output beam will stay white. If the object is birefringent then it modifies the spectral polarization fan. For example, a particle with phase retardation ~10º and the slow axis at 45º will add ~5º to the red polarization ellipticity angle and subtract ~5º from the green ellipticity angle. The red component will have the right circular polarization with ellipticity angle 45º, and it will be extinguished completely by the left circular polarizer. The green component will have ellipticity angle ~40º, and its transmission will be increased in ~4 times. So, the birefringent particle will be mostly green. If the particle or the spectral polarization fan is rotated by 90º, the picture becomes complementary and the birefringent particle is mostly red. In principle we can consider this device as a spectral Brace-Kohler compensator, which rotates the polarization ellipse spectrally rather than mechanically.

In order to generate the spectral fan of polarization ellipses one can use a waveplate, which is made of uniaxial gyrotropic crystal, such as quartz. The waveplate is cut perpendicular or near perpendicular to the crystal optical axis [3]. When the linearly polarized light propagates along the optical axis of the gyrotropic crystal the polarization plane rotates by some angle. The rotation angle is linearly proportional the waveplate thickness and inversely proportional to the wavelength. We used a waveplate with a thickness of about 8mm. The eigen polarizations are circular, and the polarization rotation angle equals to half of the phase shift between the eigen polarizations. When the beam is slightly tilted then its polarization is also affected by linear birefringence. In this case the eigen polarizations are elliptical, and the ellipticity is inverse to the angle between crystal optical axis and beam direction.

A dependence of hue on slow axis orientation was determined in the following experiment. We made a test waveplate with retardance 30nm by crossing two retardation films with retardances 110nm and 140nm (Nitto Denko, Japan). The test waveplate was placed in PPM and rotated from 0º to 360º with step 20º. Eighteen images of the 30-nm retardance test waveplate at different orientation are shown on the part (b). The initial slow axis orientation was horizontal. Experimental data and linear approximation of dependence of hue on slow axis orientation is presented in (c), the hue bar is shown on the right in color. This plot (c) depicts the hue at each orientation in a range from 0º to 180º. We used an average hue of two orientations with

difference 180º. As one could see the dependence of hue on the slow axis orientation angle φ is almost linear:

$$hue = \varphi / 180^{\circ}.$$

In additional experiments we found that the hue practically does not depend on retardance. Thus, the slow axis orientation angle can be computed in the following way:

$$\varphi = 180^{\circ} \cdot hue.$$

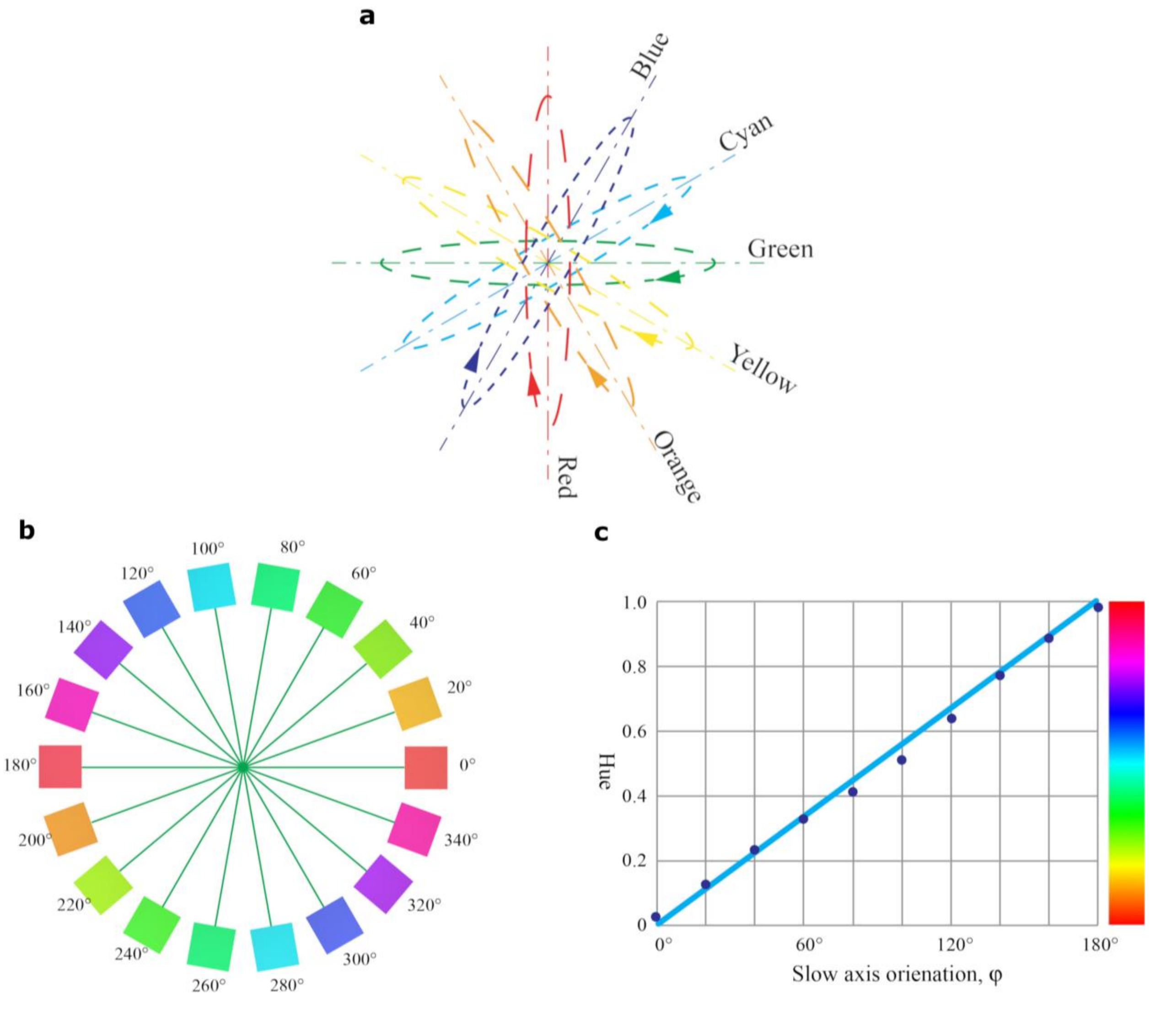

Supplementary Figure 2.

The IBI were also studied with the following contrast modalities: quantitative orientation-independent differential interference contrast (OIDIC), phase contrast (PhC) and fluorescence microscopy. The OIDIC generates two types of related images [4] 1. Gradient OIDIC is a computed color map where brightness and hue represent the magnitude and the direction of the phase gradient vector, respectively. It enables visualization of boundaries between areas with different refractive indices, such as cell walls. 2. Phase OIDIC is obtained by 2D integration of the gradient OIDIC image. The phase OIDIC image is a grayscale map, where the intensity is linearly proportional to phase (optical path length or dry mass). It provides high-resolution visualization of chromosomes, organelles, fibers, etc. In general, the OIDIC technique does not work in presence of highly birefringent objects. They destroy the two-beam interference and the image becomes strongly disrupted. This disruption is especially visible in gradient OIDIC image, as a cluster of bright colored spots enabling detection of the IBI.

An IBI in unstained deparaffinized section of a renal tubule. (a) conventional DIC image; (b) gradient OIDIC image; (c) phase OIDIC image; (d) fluorescence image of a section stained with Hoechst to highlight the nuclei. The IBI is shown by yellow arrows. The DIC and OIDIC images were taken with a bandpass filter 546/10 nm. The fluorescence image confirms that the IBI is located in a nucleus. *Olympus BX61* microscope, oil immersion objective *UPlanFl 100x/1.30 Oil P* and monochromatic CCD camera *Lumenera 3-1M*.

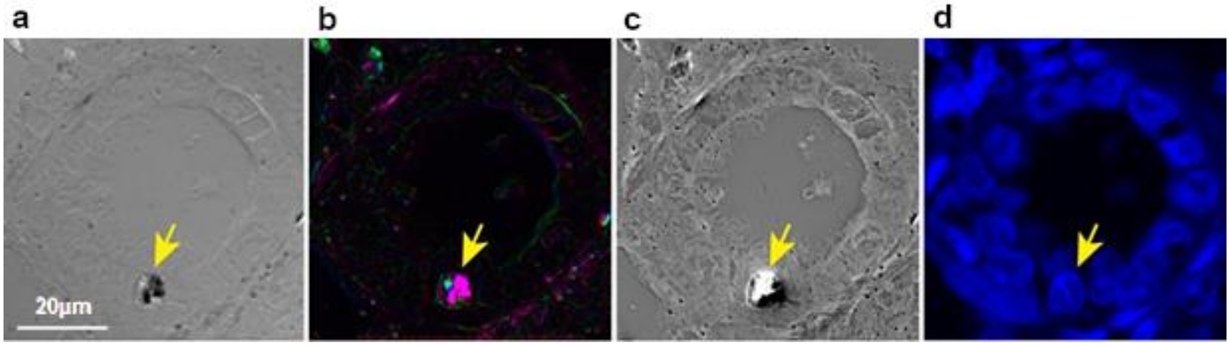

Supplementary Figure 3.

PhC has substantially lower spatial resolution than conventional DIC and OIDIC; however it is very helpful for phase imaging of birefringent objects [5]. Both the diffracted and direct (surround) waves experience the same modification of the polarization states, and therefore their mutual interference is not affected. A positive PhC objective introduces a positive quarter-wave shift between the diffracted and direct waves. In this case a particle with higher refractive index than surrounding area appears darker, and a particle with lower refractive index appears brighter. The negative PhC objective creates an inverse picture. The figure displays images of IBI taken with PPM (a), positive PhC (b) and the computed negative PhC (c). We used aqueous mounting media - Kaiser's glycerol gelatin 109242 (https://www.emdmillipore.com) with a refractive index around 1.5 [6]. Therefore the positive PhC image (b) shows the IBI significantly brighter than the surrounding area. As can be seen in the PPM image (a), most IBI have a round shape with a diameter of about 6-7 µm. Each IBI is a cluster of randomly oriented birefringent granules with size 1-2 µm. This difference in brightness is more apparent in the computed negative PhC image, which is obtained by inversion of the original image with *ImageJ* (https://imagej.net/). The sample was illuminated by white light with no spectral filters employed. In order to switch between PPM and PhC modalities we swapped a polarizer and a phase annulus in the condenser. The IBI are shown by yellow arrows. Blue arrows depict the "empty" pores. Inverted microscope *Olympus IX81* equipped with positive PhC objective *UPlanFl 100x/1.0 Oil Ph3* and *Olympus* color CCD camera *DP73*.

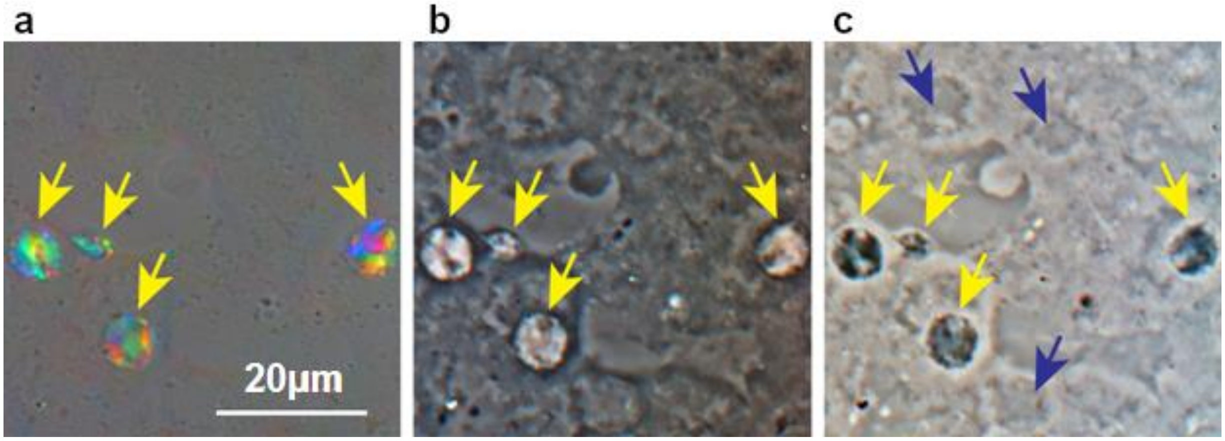

Supplementary Figure 4.

Examples of IBI signals observed with PPM in different tissues – endometrium (a, b), kidney (c, d) and liver (e, f). Here we used 4 µm thickness unstained deparaffinized tissue sections coverslipped with glycerol-based aqueous mounting media (Kaiser`s glycerol gelatin, *Merck*). For each tissue type the same area was imaged with two different magnifications – in the first column images (a, c, d) were taken with a *LUCPlanFLN 40x* objective, for the second column images (b, d, f) an additional 2x magnification microscope set up was used. A 10 percent increase in brightness and contrast were applied to the images for better visualization. *Olympus BX63* microscope, *DP80* camera, objective *LUCPlanFLN 40x* and PPM modality.

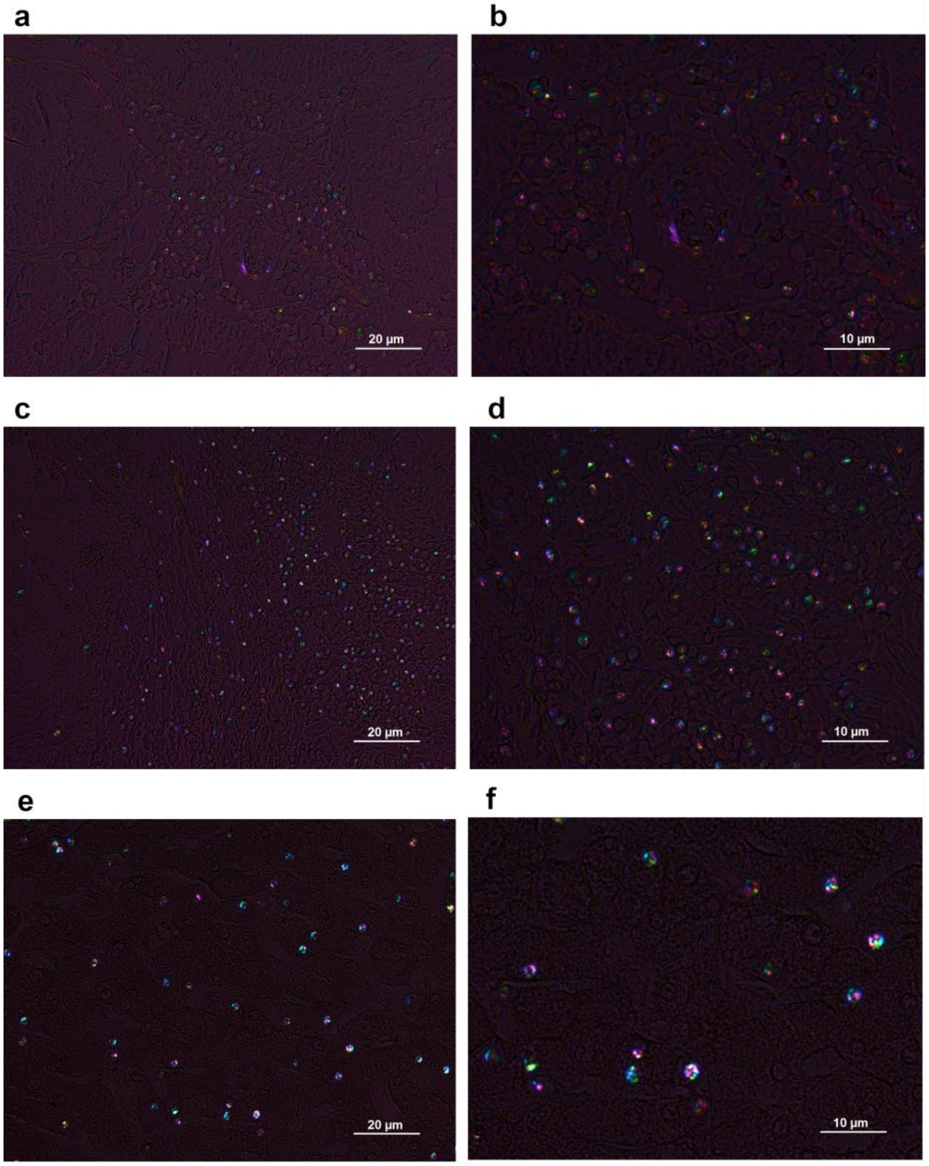

Supplementary Figure 5.

Unstained serial sections of different tissue types with varying thickness (2 µm, 4 µm and 8 µm) imaged with PPM after routine deparaffinization and coverslipping with glycerol-based aqueous mounting media. Visual representation of IBI signals amount correlation with tissue section thickness in liver (a) and kidney (b) tissue sections. A 10 percent increase in brightness and 20 percent increase in contrast were applied to the images for better visualization. *Olympus BX63* microscope, *DP80* camera, objective *LUCPlanFLN 20x* and PPM modality.

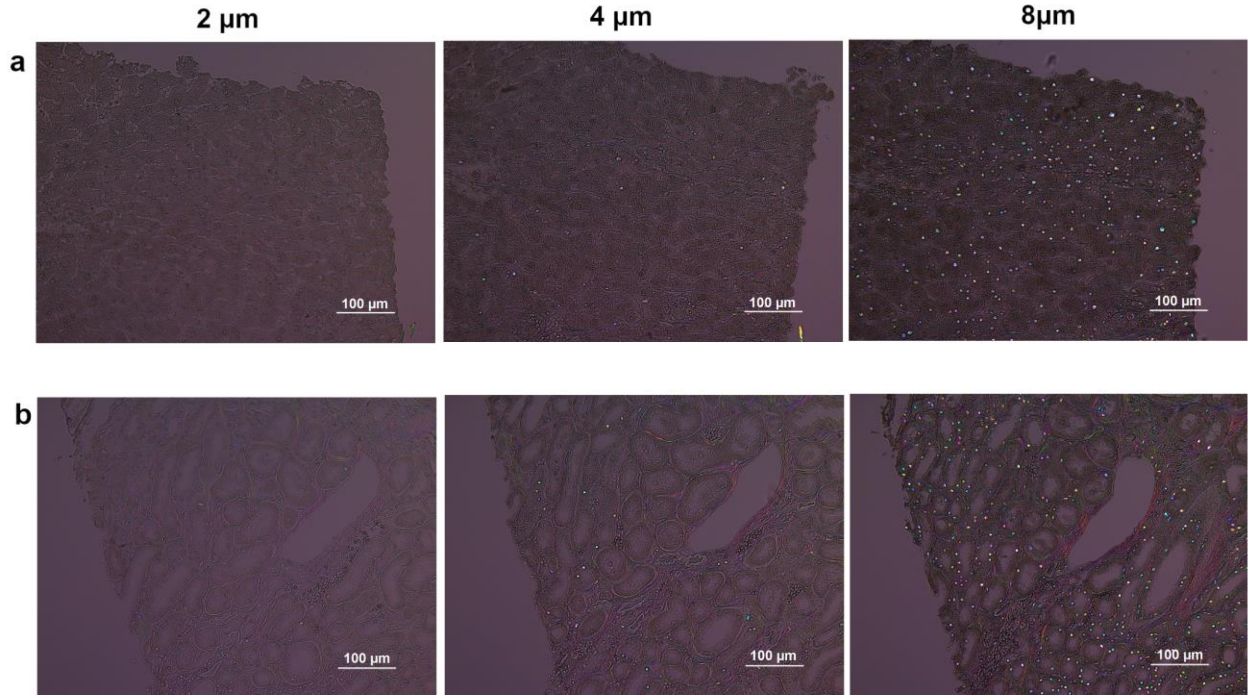

Supplementary Figure 6.

Changes in PPM signal area (a) and shape (b) of IBI during 10 cycles of heating up to 60^{0}C temperature and cooling down a deparaffinized, unstained and glycerol-coverslipped tissue section. After each melting-recrystalizing cycle the same tissue section was imaged with PPM and the same 14 IBI were identified. Each IBI was extracted from the PPM image to compare their area and shape after each cycle. Except for some random variance, no obvious trend in area or shape factor of the IBI signals was observed. The area and perimeter was evaluated based on number of segmented pixels, the shape factor was calculated from the area and perimeter ratio based on the formula: $\frac{4\pi a}{p^2}$, where a – area; p – perimeter. For the microscope table heating we used a *Haltronic* switching power supply *HPS305D*. *Olympus BX63* microscope, *DP80* camera, objective *Olympus Ach 60x* and PPM modality.

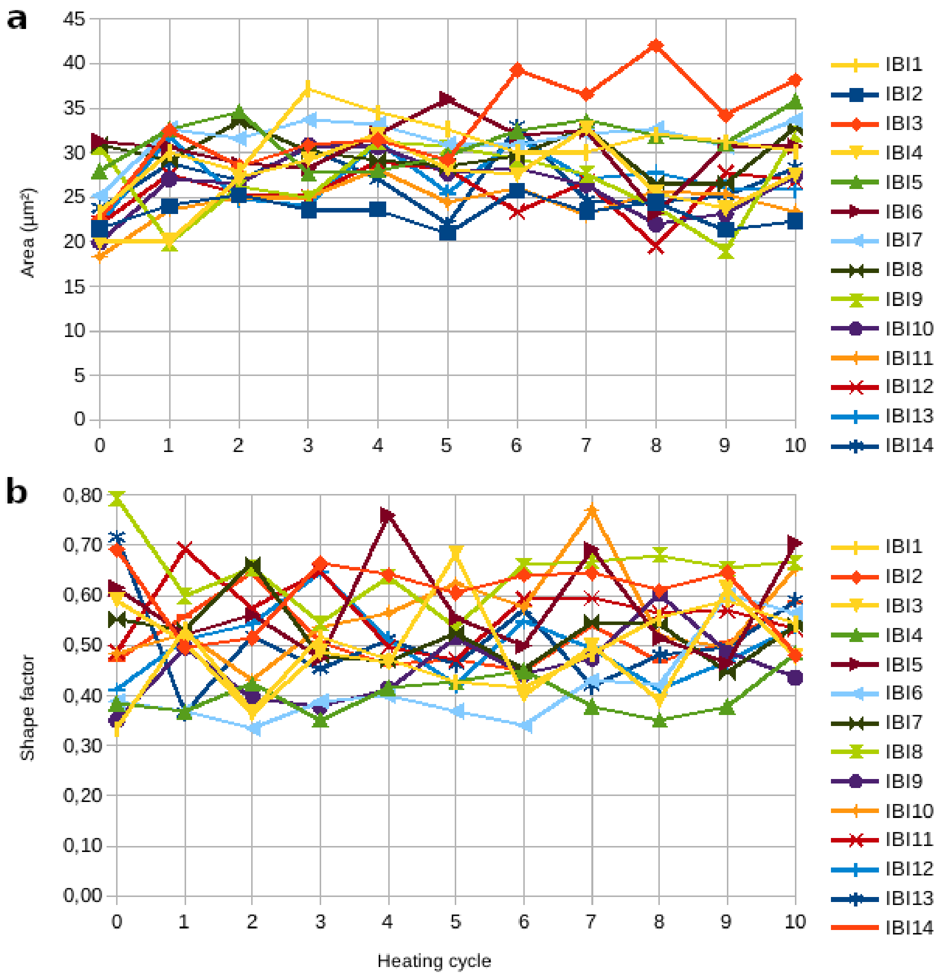

Supplementary Figure 7.

Examples of images of peritumoral inflammatory infiltrate of renal cell carcinoma used for the digital analysis for TFIIE factor and IBI associations. (a) IBI and collagen signals using PPM in deparaffinized Hoechst-stained 4 µm thick tissue section; (b) Hoechst-stained renal cell carcinoma image using DAPI fluorescence filter overlaid with extracted IBI PPM signals represented in pink; (c) The same renal cell carcinoma section slide area stained for TFIIE IHC marker with red chromogen for visualization of positive cells, bright field microscopy; (d) Final image generated for digital analysis by combining images (b) and (c), used for positive TFIIE and IBI cells counting, where blue represents Hoechst-stained nuclei, pink – IBI PPM signals, green – positive TFIIE IHC reaction. TFIIE positive nuclei were extracted from image (c) by ImageJ after translation to the PCA color space and then superimposed onto image (b). The same field of view was imaged with all three microscopy modalities (PPM, bright field and fluorescence) using *CellSens* acquisition software and processed by *ImageJ* for automated nuclei counting. Images of type (d) were used for counting IBI, nuclei and intersection (double positives)in the different samples by *ImageJ*. Numerical results are presented in supplementary table 2. *Olympus BX63* microscope, *DP80* camera, objective *LUCPlanFLN 40x* and PPM modality. The fluorescence filter cube used for DAPI fluorescence was *Olympus U_FUNA*.

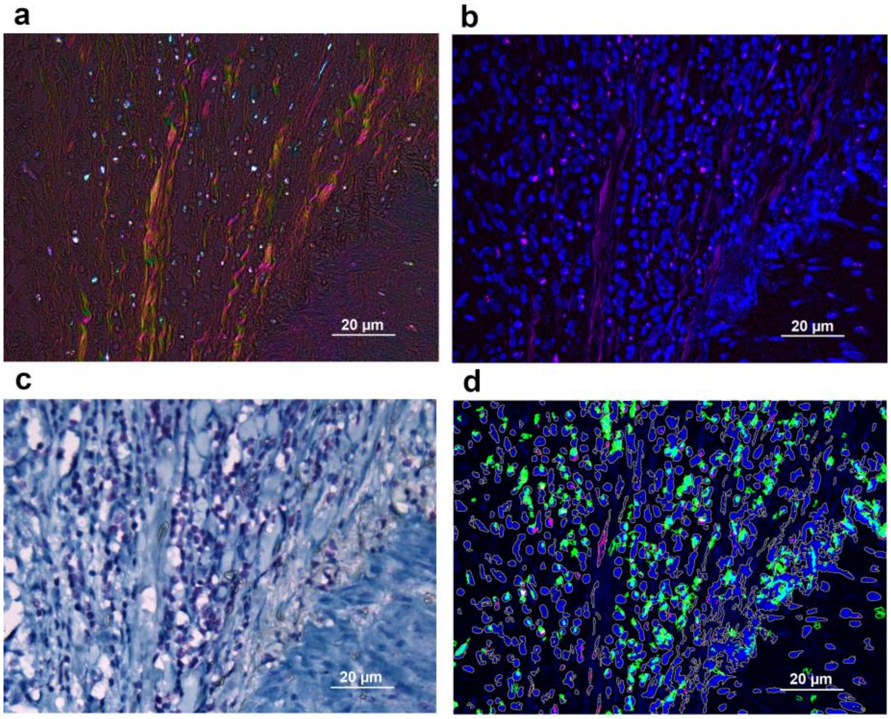

Supplementary Table 1.

Heating experiment to examine the properties of the IBI melting and recrystallization process. A deparaffinized unstained glycerol-coverslipped 4µm thick slide of hepatocellular carcinoma tissue was placed on a heating table under the microscope at room temperature. While the slide was heated we registered temperatures when the first and the last IBI disappeared in the field of view. The heating table was switched off after all IBI disappeared; then the temperatures were registered when the first and the last IBI reappeared. There were pauses between the heating cycles for the specimen to cool down to 30°C before starting another heating cycle as described above. The field of view was imaged before and after each heating cycle, without moving the slide; the imaging data were used for Supplementary Figure 5 to assess the change of IBI structure during the recrystallization process. This experiment revealed that the temperature when IBI signals started to disappear was similar to the melting point of paraffin wax used in tissue processing procedures. For the microscope table heating we used a *Haltronic* switching power supply *HPS305D*. The temperature was measured manually using an infrared thermometer *UNI-T UT300A*. *Olympus BX63* microscope, *DP80* camera, objective *LUCPlanFLN 40x* and PPM modality.

Temperature measurements (°C)				
Heating cycle	First IBI disappeared	All IBI disappeared	First IBI reappeared	All IBI reappeared
1	59	61	55	40
2	60	62	48	38
3	48	60	46	36
4	49	50	47	37
5	48	50	46	36
6	-	50	45	36
7	48	49	45	36
8	49	53	46	35

9	46	48	45	36
10	47	49	46	37
Mean	50.4	53.2	46.9	36.7
Sd	5.2	5.6	3	1.4
Median	48	50	46	36

Supplementary Table 2.

Summary of results from digital analysis of the TFIIE factor and IBI associations. For each case we examined 20 fields of view using *Olympus LUCPlanFLN 40x* objective as described in Supplementary Fig. 4. Nuclei containing IBI (IBI+ nuclei), positive for TFIIE IHC (TFIIE+ nuclei) and double positive (THIIE+IBI+ nuclei) were enumerated. The proportion of double positive (THIIE+IBI+ nuclei) was calculated for each case.

Case	IBI+ nuclei	TFIIE+ nuclei	TFIIE+IBI+ nuclei	TFIIE+IBI+/IBI+ nuclei, %
Renal cell carcinoma				
E2726	1216	2375	470	39
E2731	545	1617	170	31
E2732	1215	1459	236	19
E2734	897	1206	252	28
Hepatocellular carcinoma				
E2736	1241	701	81	7
E2739	858	864	44	5
E2774	813	2057	221	27
E2779	400	4245	358	90

Supplementary Video 1.

Re-crystallization of IBI in liver tissue section recording. A paraffin section of liver tissue stained with hematoxylin for nuclei visualization was heated up to 60^{0}C until the IBI melted, and then videorecorded in the process of cooling down. For the microscope table heating we used *Haltronic* switching power supply *HPS305D*. *Olympus BX63* microscope, *DP80* camera, objective *LUCPlanFLN 40x* and PPM modality.

References

1. Shribak, M., *Polychromatic polarization microscope: bringing colors to a colorless world.* Scientific Reports, 2015. **5**(1): p. 17340.
2. Shribak, M., *Polarization.* Handbook of Optical Metrology: Principles and Applications (ed. T. Yoshizawa), CRC Press, Boca Raton, FL, USA, 2009. pp. 339-350 .
3. Shribak, M., *Use of gyrotropic birefringent plate as quarter-wave plate*. Soviet Journal of Optical Technology, 1986. **53**(8): p. 443-446.
4. J.E. Malamy, M. Shribak, *High resolution imaging of epithelial cell migration and wound healing in a Cnidarian model using an orientation-independent DIC microscope*. Journal of Microscopy, 2018. **270**(3): p. 290-301.
5. R.Oldenbourg, and M.Shribak, *Microscopes*. Handbook of Optics, Third Edition, Volume I: Geometrical and Physical Optics, Polarized Light, Components and Instruments (ed. Michael Bass), McGraw-Hill Professional, 2010.
6. https://www.chemicalbook.com/ChemicalProductProperty_EN_CB2854418.htm (accessed on 11 November 2020).
